# Limited impact of NLRP3 inflammasome deficiency on diet-induced metabolic dysfunction-associated steatohepatitis in mice

**DOI:** 10.1016/j.bbrep.2026.102811

**Published:** 2026-09-23

**Authors:** Daisuke Taguchi, Yohei Shirakami, Toshihide Maeda, Daisuke Minowa, Masaya Kubota, Hiroyasu Sakai, Hiroyuki Tomita, Masahito Shimizu

**Affiliations:** a Department of Gastroenterology, Gifu University Graduate School of Medicine, 1-1 Yanagido, Gifu, 501-1194, Japan; b Department of Tumor Pathology, Gifu University Graduate School of Medicine, 1-1 Yanagido, Gifu, 501-1194, Japan

**Keywords:** Inflammasome, Metabolic dysfunction-associated steatohepatitis, Metabolic dysfunction-associated steatotic liver disease, NLRP3, Steatotic liver disease

## Abstract

Metabolic dysfunction-associated steatohepatitis (MASH) is a progressive form of metabolic dysfunction-associated steatotic liver disease (MASLD) and a growing cause of chronic liver disease worldwide. Although the NLRP3 inflammasome has been implicated in MASH pathogenesis, its contribution remains controversial across experimental models. We investigated whether systemic deletion of NLRP3 attenuates diet-induced MASH using a choline-deficient, l-amino acid-defined, high-fat diet (CDAHFD) model. Wild-type (WT) and NLRP3-knockout (KO) mice were fed either CDAHFD or a control diet. CDAHFD induced comparable hepatic steatosis, inflammation, fibrosis, and serum alanine aminotransferase elevation in both genotypes. Systemic and hepatic oxidative stress was also comparable between WT and NLRP3-KO mice. Total NAFLD activity scores did not differ between the two genotypes. Although hepatic *Adgre1* expression showed a partial reduction in NLRP3-KO mice, pro-inflammatory cytokines and fibrosis-related genes were not significantly altered. These findings indicate that systemic NLRP3 deletion does not substantially attenuate CDAHFD-induced MASH. Together with recent reports using other diet-induced MASH models, our results support the concept that global NLRP3 deficiency alone is insufficient to prevent MASH progression and highlight the context-dependent role of inflammasome signaling in steatohepatitis.

## Highlights

• NLRP3 deficiency was examined in a diet-induced mouse model of MASH.
• NLRP3 loss did not significantly improve steatosis or liver injury.
• Fibrogenic gene expression was largely unchanged in NLRP3-deficient mice.
• Systemic NLRP3 suppression alone may have limited therapeutic benefit in MASH.

## Graphical abstract

**Figure:**
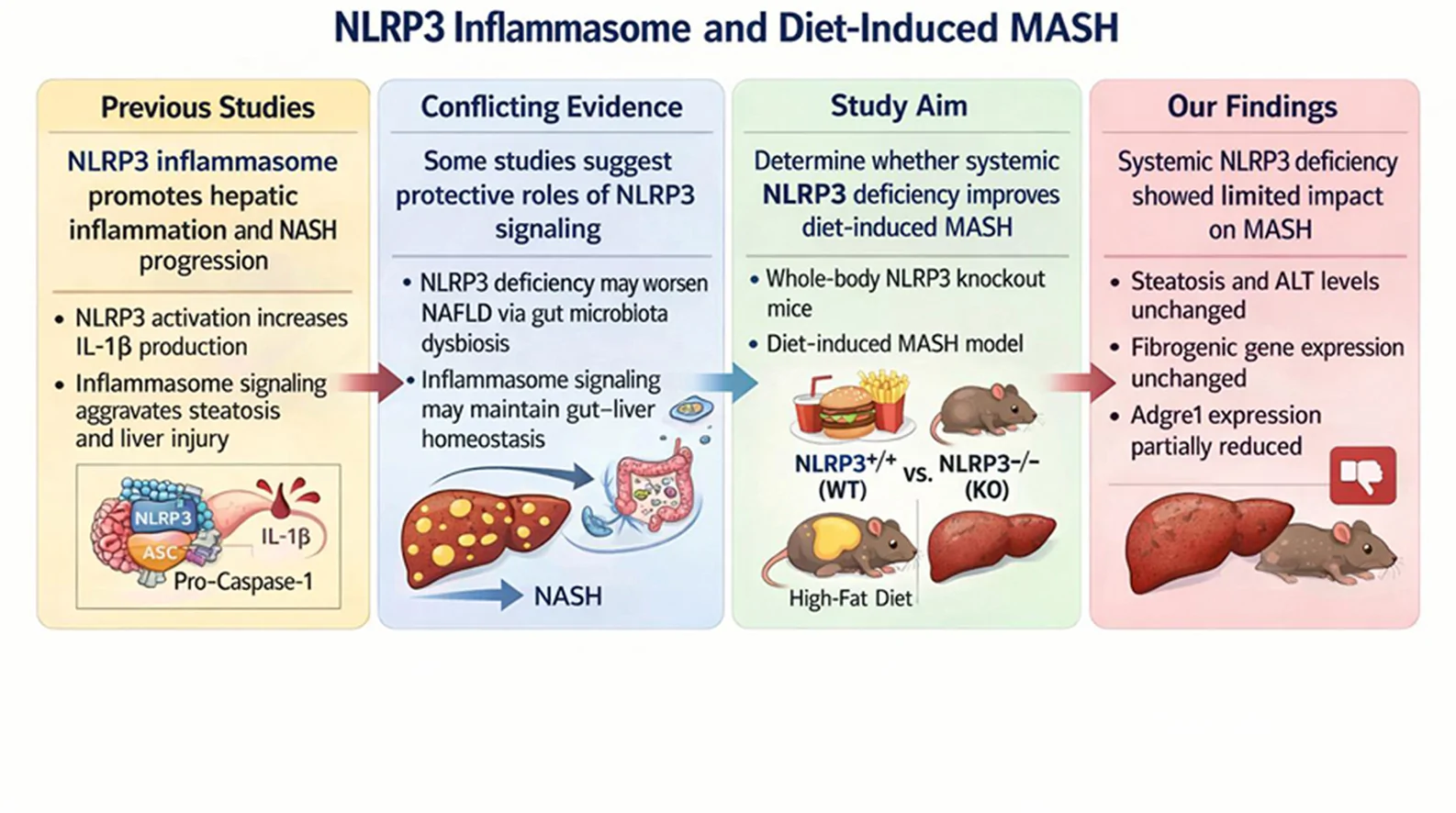

## 1 Introduction

Metabolic dysfunction-associated steatotic liver disease (MASLD) is the most prevalent chronic liver disease worldwide and is strongly associated with obesity, type 2 diabetes, and other cardiometabolic risk factors [[1], [2], [3], [4]]. A progressive subset of MASLD develops into metabolic dysfunction-associated steatohepatitis (MASH), characterized by hepatocellular ballooning and lobular inflammation, which increases the risks of fibrosis, cirrhosis, and liver-related mortality [1,5]. In addition, MASLD is associated with increased cardiovascular risk, distinguishing its natural history from that of other chronic liver diseases [[6], [7], [8]]. Although lifestyle modification remains the cornerstone of therapy [1,9,10], adherence is often limited, and effective pharmacological strategies are still needed.

The Nod-like receptor protein 3 (NLRP3) inflammasome is a key regulator of innate immune responses and has been implicated in MASH pathogenesis [11,12]. Its activation promotes caspase-1–mediated maturation of interleukin-1β and interleukin-18, leading to inflammatory cell recruitment and hepatocellular injury [12]. Increased NLRP3 expression has been reported in patients with MASH [13], suggesting that inflammasome signaling may represent a therapeutic target. However, experimental studies evaluating NLRP3 inhibition in dietary MASH models have yielded inconsistent results depending on the dietary model, disease stage, and experimental conditions [[14], [15], [16], [17], [18], [19]]. In addition, recent studies have suggested that global NLRP3 deficiency does not substantially protect against diet-induced MASH, highlighting the need for independent validation across different experimental settings.

Therefore, the present study aimed to evaluate the impact of global NLRP3 deficiency in a choline-deficient, l-amino acid-defined, high-fat diet (CDAHFD)-induced MASH model [20]. By examining hepatic steatosis, inflammation, oxidative stress, and fibrosis, we sought to determine whether the previously reported phenotype could be reproduced in an independent dietary model.

## 2 Material and methods

### 2.1 Animals and diets

Male C57BL/6 J mice and NLRP3-KO mice with a C57BL/6 J background, B6·129S6-*Nlrp3*^*tm1Bhk*^/J (Strain 021302), aged 8 weeks, were obtained from Jackson Laboratory (Bar Harbor, ME, USA). Animals were maintained in plastic enclosures under conditions regulated for humidity (50% ± 10%), light (12-h light/dark cycle), and temperature (23°C ± 2°C), with free access to water and diet. MASH was induced in mice by administering a CDAHFD (#A06071302, Research Diets Inc., New Brunswick, NJ, USA), as previously described [20]. Control animals were fed a control diet (#A06071314, Research Diets Inc.).

### 2.2 Experimental protocol

The animals were divided into four groups: group 1, wild-type (WT) mice fed a control diet (*n* = 4); group 2, NLRP3-KO mice fed a control diet (*n* = 3); group 3, WT mice fed CDAHFD (*n* = 5); and group 4, NLRP3-KO mice fed CDAHFD (*n* = 4). The study began after a one-week acclimatization period, and the actual experimental duration, feeding control diet or CDAHFD, was 9 weeks. All mice were euthanized at 18 weeks of age, and blood samples and organs were collected (Fig. 1A). All experimental procedures were conducted in accordance with the National Institutes of Health Guide for the Care and Use of Laboratory Animals and Animal Care Guidelines established by the Animal Care and Use Committee of Gifu University (Gifu, Japan). The study protocol was reviewed and approved by the Institutional Animal Experiments Committee of Gifu University (approval number: AG-P-C-20230011).

**Fig. 1:**
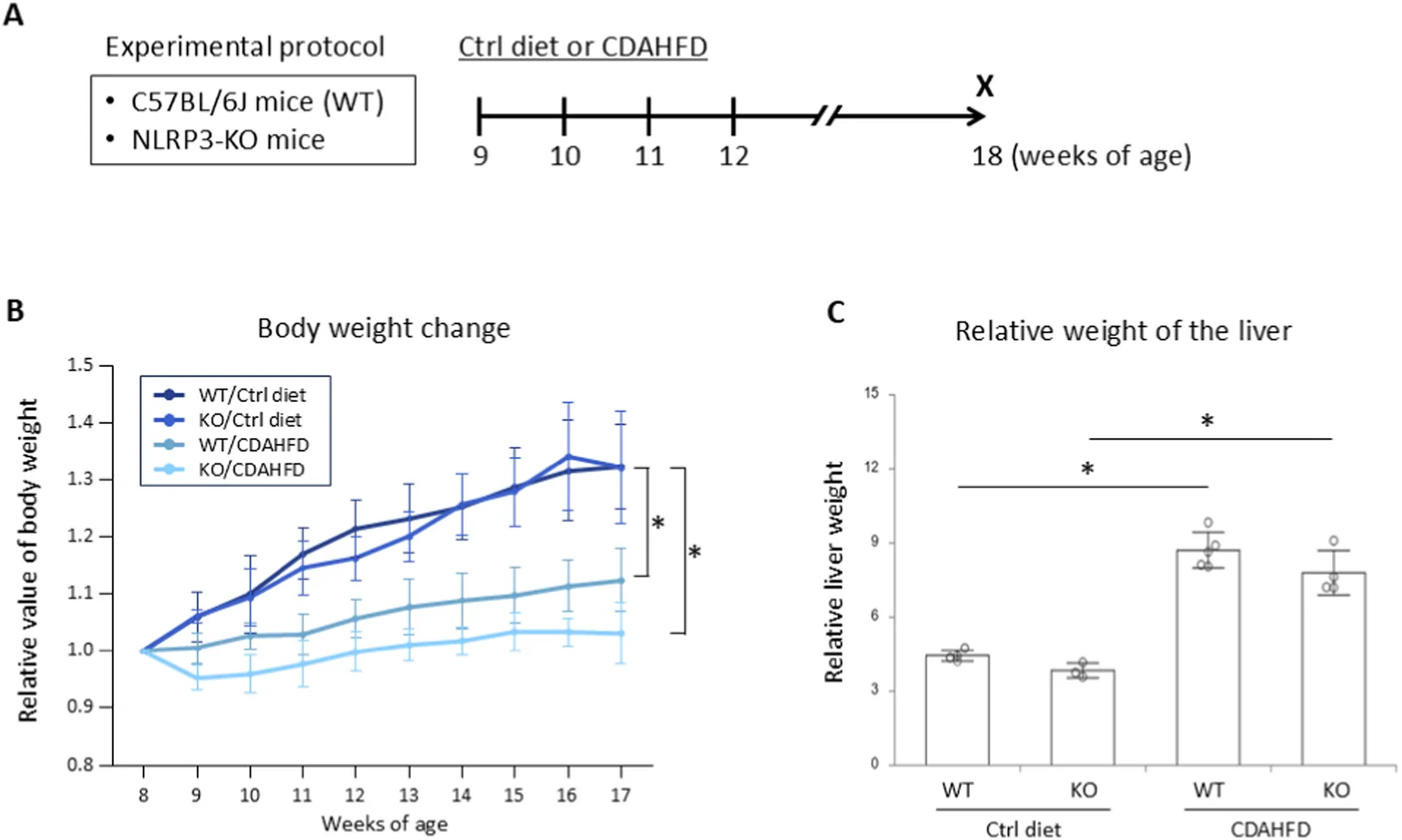
**Experimental protocol and general observation.** (A) Wild-type (WT) and NLRP3-KO (KO) mice were fed a control (Ctrl) diet or a metabolic dysfunction-associated steatohepatitis-inducing diet (choline-deficient, l-amino acid-defined, high-fat diet, CDAHFD) from 9 to 18 weeks of age. (B) Body weight was monitored throughout the experimental period. CDAHFD feeding resulted in a lower increase in body weight than the Ctrl diet in both WT and KO mice. (C) Relative liver weight was measured at the end of the experiment. CDAHFD feeding significantly increased relative liver weight in both WT and KO mice, with no significant difference between WT and KO mice fed CDAHFD. Data are presented as mean ± SD (WT/Ctrl, *n* = 4; KO/Ctrl, *n* = 3; WT/CDAHFD, *n* = 5; KO/CDAHFD, *n* = 4). Statistical analyses were performed using two-way analysis of variance and Tukey's honest significant difference test. *P* < 0.05. NLRP3, NOD-like receptor protein 3; X, euthanasia.

### 2.3 Histological assessment

Liver tissue was preserved in 10% neutral buffered formalin, processed into paraffin blocks, and sectioned for histological analysis. Hematoxylin and eosin (HE) staining was used for general histopathological assessment, and Sirius red staining was conducted to evaluate liver fibrosis. The severity of MASH, including hepatic fibrosis, was evaluated using the NAFLD scoring system [21]. Liver steatosis was assessed on HE-stained slides at 40× magnification and graded for macro-vesicular steatosis, micro-vesicular steatosis, and hypertrophy using the following categories: zero (<5%), one (5–33%), two (34–66%), and three (>66%). Additional lipid-specific staining, which may provide complementary information, could not be performed because frozen liver tissues were unavailable. Liver inflammation was assessed at 100× magnification and evaluated by counting the number of inflammatory foci per field as follows: zero (<0.5 foci), one (0.5–1.0 foci), two (1.0–2.0 foci), and three (>2.0 foci). The total NAFLD score was calculated by summing the steatosis and inflammation scores [21]. Liver fibrosis was also histologically evaluated by quantification of Sirius-red stained areas using NIH Image software (Bethesda, MD, USA) according to the section of tutorials and examples on the website of the software.

### 2.4 Laboratory assessment

Serum alanine aminotransferase (ALT) and triglyceride levels were evaluated at a commercial laboratory (SRL Inc., Tokyo, Japan). To assess oxidative stress, serum hydroperoxide concentrations were determined using derivatives of reactive oxygen metabolites (d-ROMs) with the FREE system (Diacron International srl, Grosseto, Italy), following the standard protocol.

### 2.5 Measurement of hepatic oxidative stress in the liver

Hepatic oxidative DNA damage was assessed by measuring 8-hydroxy-2′-deoxyguanosine (8-OHdG) levels. Approximately 20 mg of frozen liver tissue was homogenized, and genomic DNA was isolated using QIAamp DNA Mini Kit (QIAGEN, Hilden, Germany). Purified DNA was enzymatically hydrolyzed using Nuclease P1 (N8630, Sigma-Aldrich, St. Louis, MO, USA) followed by alkaline phosphatase treatment. The samples were subjected to a competitive enzyme-linked immunosorbent assay (ELISA) using the 8-OHdG ELISA kit (Nikken SEIL, Shizuoka, Japan). The results were normalized to the amount of DNA and expressed as ng 8-OHdG per mg DNA.

### 2.6 Gene expression assessment via RNA extraction and quantitative real-time reverse transcription PCR

Total RNA was extracted from liver tissues using an RNeasy Mini Kit (QIAGEN, Venlo, Netherlands). Complementary DNA was synthesized using a High-Capacity cDNA Reverse Transcription Kit (Applied Biosystems, Foster City, CA, USA). Quantitative real-time reverse transcription PCR was performed on a LightCycler 96 System (Roche Diagnostics, Indianapolis, IN, USA) using SYBR Green chemistry. The thermal cycling conditions were as follows: pre-incubation at 95°C for 10 min, followed by 40 cycles of denaturation at 95°C for 10 s, annealing at 60°C for 10 s, and extension at 72°C for 15 s. Gene expression levels of *Adgre1*, *Col1a1, Gapdh*, *Il1b*, *Il18*, *Timp*, and *Tnf* were assessed using specific primer sets described previously [[22], [23], [24], [25]] or as follows: *Mmp2* forward, 5‐GAG CGT GAA GTT TGG AAG CA‐3, and reverse, 5‐GAA GAT GAT AGG GCC CGT GG‐3; *Mmp7* forward, 5‐TCT AGG TCA TGC CTT CGC AC‐3, and reverse, 5‐CGT CCT CAC CAT CAG TCC AG‐3. The levels of *Gapdh* were used for normalization of gene expressions.

### 2.7 Statistical analyses

Continuous variables were analyzed using two-way analysis of variance. Multiple pairwise comparisons were performed using Tukey's honest significant difference test. Data were assessed for normality using the Shapiro–Wilk test before parametric analyses. Ordinal histological scores were analyzed using the Kruskal–Wallis test followed by Dunn's multiple-comparison test. All tests were two-sided, and *P* values < 0.05 were considered statistically significant. Statistical analyses were performed using R version 4.4.2 (R Foundation for Statistical Computing, Vienna, Austria).

## 3 Results

### 3.1 General observations

The changes in body weights and relative liver weights at the end of the experiment are shown in Fig. 1B and C, respectively. In WT mice, total body weight was significantly lower in the MASH group than in the control group. Similarly, in NLRP3-KO mice, body weight was significantly lower in the MASH group than in the control group (Fig. 1B). Relative liver weight was significantly higher in the MASH group than in the control group in both WT and NLRP3-KO mice (Fig. 1C). These results suggest that the MASH diet induced comparable systemic and hepatic effects in both WT and NLRP3-KO mice.

### 3.2 Effects of NLRP3 deficiency on liver injury and oxidative stress

Laboratory findings are shown in Fig. 2. Serum ALT levels were significantly higher in the MASH group than in the control group in both WT and NLRP3-KO mice (Fig. 2A), whereas no significant difference was observed in serum triglyceride levels among all groups. Serum d-ROM levels were significantly higher in the MASH group than in the control group in both WT and NLRP3-KO mice (Fig. 2B). Hepatic oxidative stress was also assessed by the levels of 8-OHdG, which revealed that those levels were significantly elevated in CDAHFD-fed groups than in the respective control groups (Fig. 2C). These results suggest that the MASH diet caused hepatic injury and enhanced systemic oxidative stress regardless of NLRP3 deficiency.

**Fig. 2:**
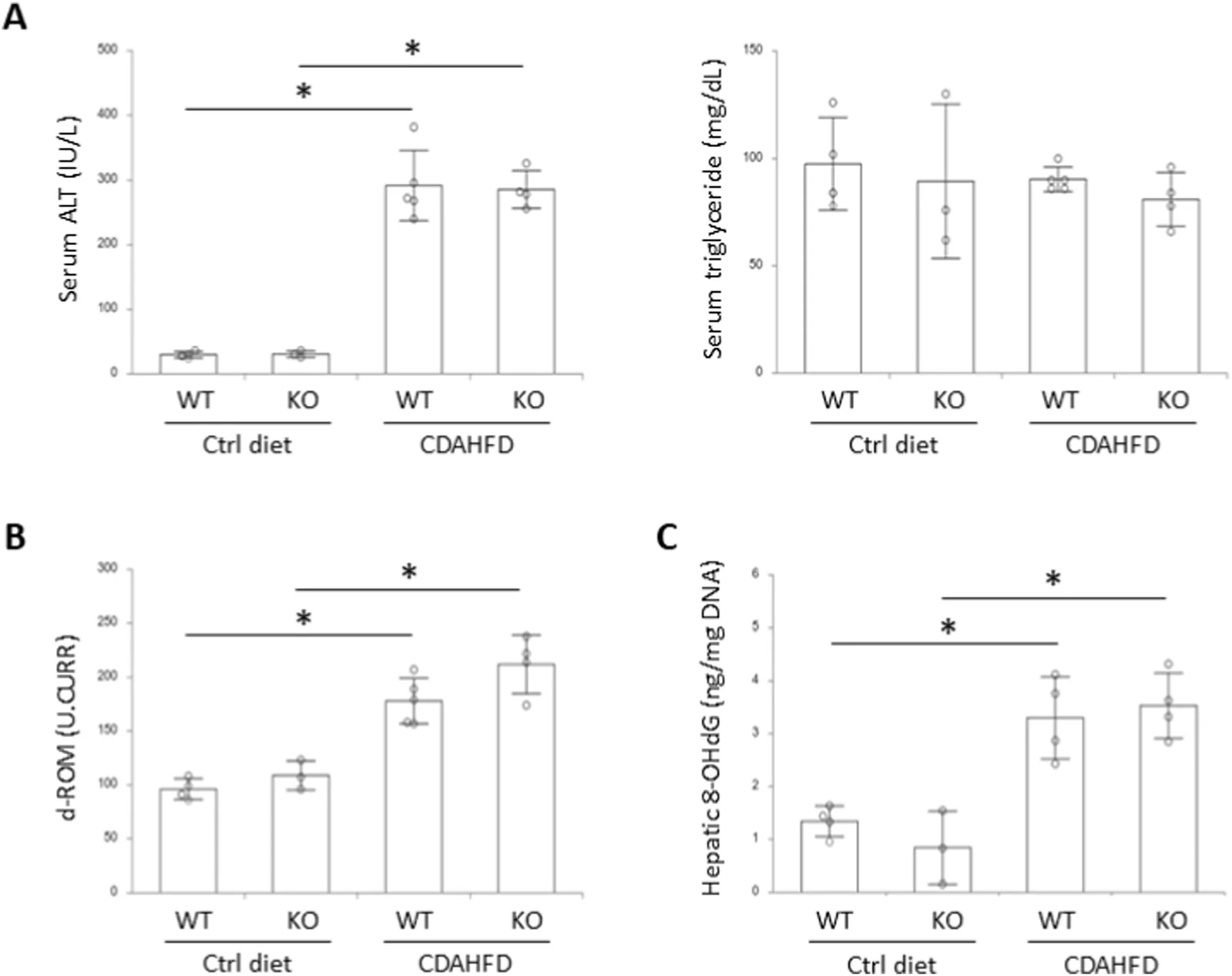
**Biochemical evaluation of serum and systemic and hepatic oxidative stress.** (A) Serum alanine aminotransferase (ALT) and triglyceride levels were measured. Choline-deficient, l-amino acid-defined, high-fat diet (CDAHFD) feeding significantly increased serum ALT levels in both WT and KO mice, whereas serum triglyceride levels were not significantly altered. (B) Systemic oxidative stress was evaluated by measuring serum derivatives of reactive oxygen metabolites (d-ROMs). CDAHFD feeding significantly increased serum d-ROM levels in both WT and KO mice. (C) Hepatic oxidative stress was assessed by measuring 8-hydroxy-2′-deoxyguanosine (8-OHdG) using an ELISA kit. CDAHFD feeding significantly increased hepatic 8-OHdG levels in both WT and KO mice. NLRP3 deficiency did not significantly affect these biochemical or oxidative stress parameters in CDAHFD-fed mice. Data are presented as mean ± SD (WT/Ctrl, *n* = 4; KO/Ctrl, *n* = 3; WT/CDAHFD, *n* = 5; KO/CDAHFD, *n* = 4). Statistical analyses were performed using two-way analysis of variance and Tukey's honest significant difference test. *P* < 0.05. Ctrl, control; KO, NLRP3-KO mice; WT, wild-type mice.

### 3.3 Histological assessment of steatosis, inflammation, and fibrosis

Histological evaluation of HE- and Sirius red-stained liver sections is shown in Fig. 3. The CDAHFD-fed group developed more severe steatosis and fibrosis than that of the control group in both WT and NLRP3-KO mice (Fig. 3A). The CDAHFD group showed significantly greater macro-vesicular and micro-vesicular steatosis in both WT and NLRP3-KO mice, with no significant difference in hypertrophy (Fig. 3B). Liver inflammation was significantly greater in the CDAHFD-fed group than in the control group in both WT and NLRP3-KO mice. The total NAFLD score was significantly higher in the CDAHFD-fed group than in the control group in both genotypes, whereas no significant difference was found between WT and NLRP3-KO mice. Liver fibrosis was evaluated by histological scoring system as well as quantification of Sirius red-stained area. Liver fibrosis was also much more severe in the CDAHFD group compared to respective control group, but no significant difference was observed between WT and KO (Fig. 3C and D). These findings suggest that NLRP3 deficiency had limited impact on the histological features of MASH under the present experimental condition.

**Fig. 3:**
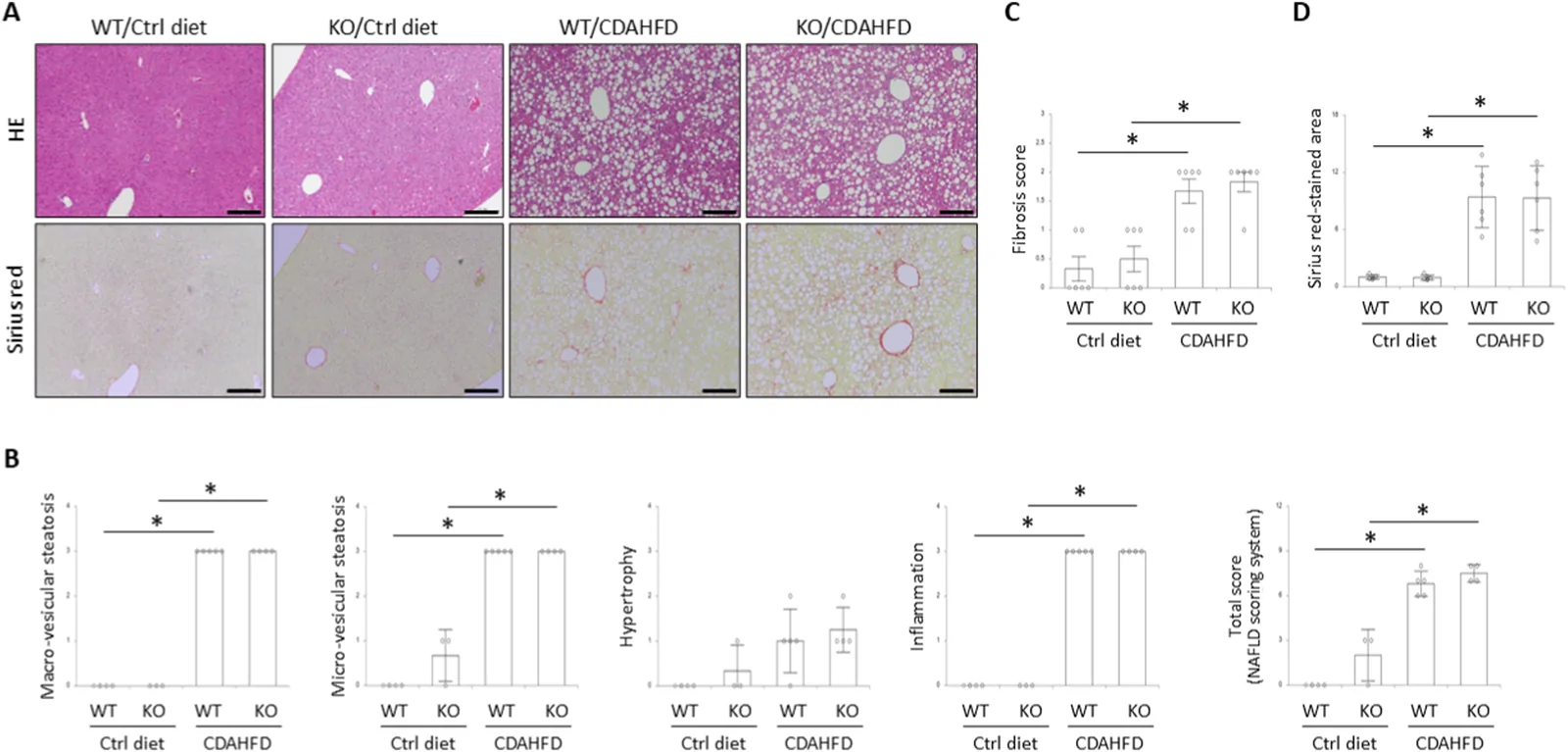
**Effects of NLRP3 deficiency on diet-induced metabolic dysfunction-associated steatohepatitis.** (A) Histopathological alterations in the liver were evaluated by hematoxylin and eosin (HE) and Sirius red staining. Scale bars, 200 μm. (B) Macrovesicular steatosis, microvesicular steatosis, hepatocellular hypertrophy, and inflammation were evaluated according to the NAFLD scoring system, and the total score was calculated. (C) Hepatic fibrosis was evaluated using the scoring system. (D) The Sirius red-positive area was quantified and expressed as a relative value. Choline-deficient, l-amino acid-defined, high-fat diet (CDAHFD) feeding markedly increased hepatic steatosis, inflammation, and fibrosis-related histopathological scores and Sirius red-positive areas. NLRP3 deficiency did not significantly ameliorate CDAHFD-induced hepatic steatosis, inflammation, or fibrosis. Data are presented as mean ± SD (WT/Ctrl, *n* = 4; KO/Ctrl, *n* = 3; WT/CDAHFD, *n* = 5; KO/CDAHFD, *n* = 4). Ordinal histological scores were analyzed using the Kruskal–Wallis test followed by Dunn's multiple-comparison test. Continuous variables were analyzed using two-way analysis of variance followed by Tukey's honest significant difference test. *P* < 0.05. Ctrl, control; KO, NLRP3-KO mice.

### 3.4 Hepatic expression of fibrosis- and inflammation-related genes

The mRNA expressions of *Col1a1* and *Timp1* in the liver were significantly higher in the CDAHFD group than in the control group in WT mice (Fig. 4A). Similar increases were observed in NLRP3-KO mice. The expression levels of *Mmp2* and *Mmp7* tended to be higher in the MASH groups compared to those respective controls, but there were no significant differences. The expression of *Adgre1* was significantly elevated in the CDAHFD group compared with the control group in WT mice. In NLRP3-KO mice, *Adgre1* expression was also increased by CDAHFD feeding; however, its expression level was significantly lower than that in WT mice fed CDAHFD (Fig. 4B). Similarly, hepatic *Tnf* expression was significantly increased in the CDAHFD group compared with the control group in WT mice. In NLRP3-KO mice, *Tnf* expression showed an increasing trend following CDAHFD feeding, although no significant difference was observed between WT and NLRP3-KO mice in the CDAHFD group (Fig. 4B). The expressions of *Il1b* tended to be lower in NLRP3-KO mice, but there was no significant difference between WT and NLRP3-KO mice. There was no significant difference in the levels of *Il18* among groups. These findings suggest that CDAHFD feeding induced hepatic fibrosis- and inflammation-related gene expression, and that NLRP3 deficiency partially attenuated inflammation-associated gene expression in this study.

**Fig. 4:**
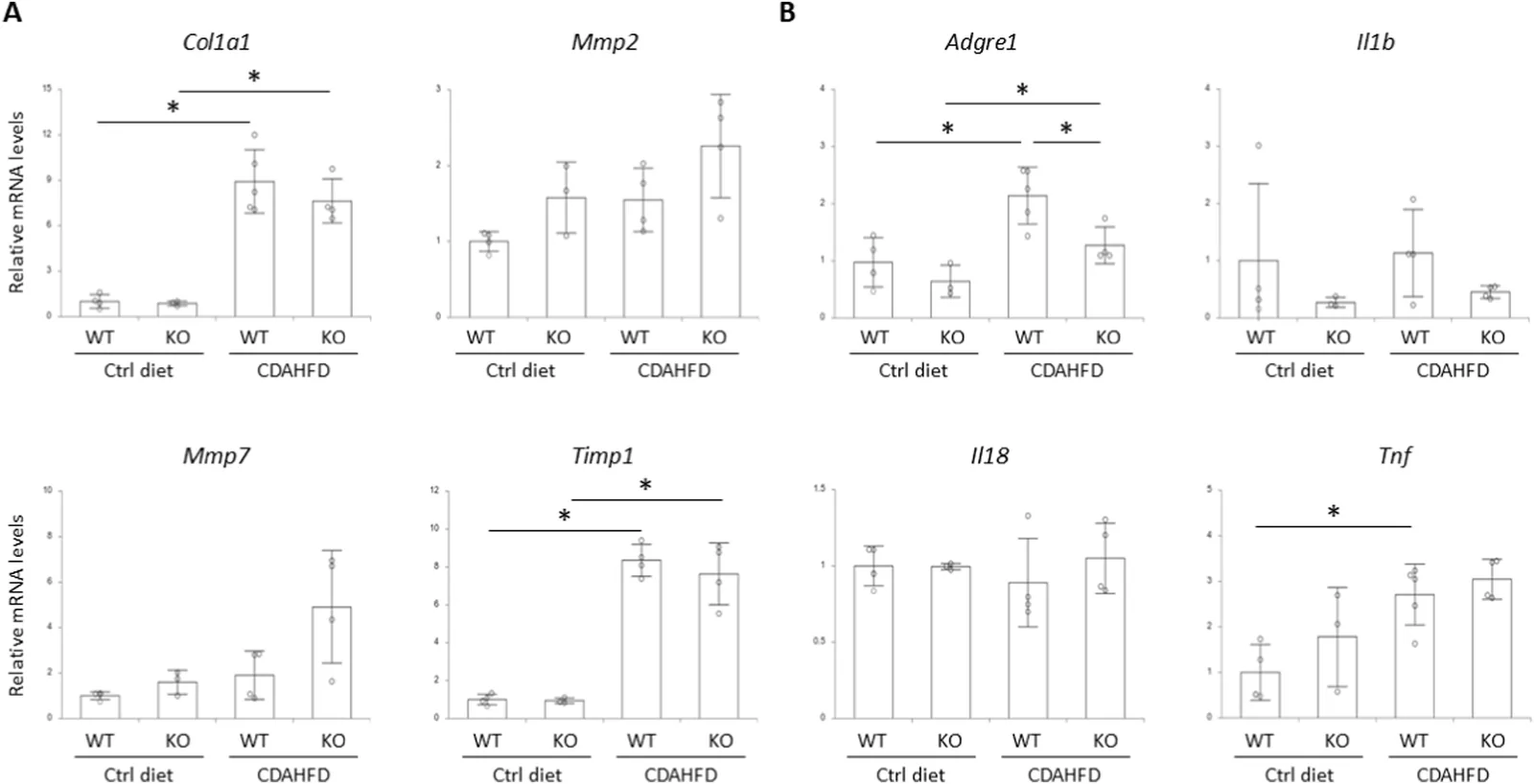
**Effects of NLRP3 deficiency on gene expressions in the liver.** (A and B) Relative hepatic mRNA expression levels were evaluated by quantitative real-time reverse transcription PCR. Choline-deficient, l-amino acid-defined, high-fat diet (CDAHFD) feeding significantly increased the hepatic expression of *Col1a1* and *Timp1* in both WT and KO mice. In contrast, *Mmp2*, *Mmp7*, *Il1b*, and *Il18* expression levels were not significantly altered by either CDAHFD feeding or NLRP3 deficiency. CDAHFD feeding increased *Tnf* expression in WT mice. Notably, *Adgre1* expression was significantly increased by CDAHFD feeding in WT mice and was significantly lower in NLRP3-KO mice than in WT mice following CDAHFD feeding. Data are presented as mean ± SD (WT/Ctrl, *n* = 4; KO/Ctrl, *n* = 3; WT/CDAHFD, *n* = 5; KO/CDAHFD, *n* = 4). Statistical analyses were performed using two-way analysis of variance followed by Tukey's honest significant difference test. *P* < 0.05. Ctrl, control; KO, NLRP3-KO mice; WT, wild-type mice.

## 4 Discussion

Although increasing evidence suggests that activation of the NLRP3 inflammasome contributes to hepatic inflammation and fibrosis in MASH [12], the pathological significance of genetic NLRP3 deficiency in CDAHFD-induced MASH has not been fully clarified. The present study demonstrates that systemic deletion of NLRP3 did not substantially ameliorate CDAHFD-induced MASH. Both WT and NLRP3-KO mice developed comparable steatosis, inflammation, fibrosis, and elevations in serum ALT and oxidative stress markers. Total NAFLD activity scores and fibrosis-related gene expression were not significantly different between genotypes under CDAHFD feeding. Although hepatic *Adgre1* expression was reduced in NLRP3-KO mice, the magnitude of this effect was modest and was not accompanied by clear improvements in histological severity or biochemical injury. These findings suggest that global NLRP3 deletion exerts, at most, limited effect sizes in this dietary MASH model.

Previous studies investigating NLRP3 inhibition in metabolic liver disease have yielded inconsistent results. Some reports indicate that NLRP3 deficiency exacerbates gut–liver axis dysregulation and worsens steatohepatitis [14,15], whereas others show no significant attenuation of diet-induced NASH following genetic deletion or pharmacologic inhibition of NLRP3 [16,17]. Conversely, NLRP3 gain-of-function models exhibit hepatic inflammation and fibrosis resembling human MASH [26,27], and cell-specific deletion in myeloid cells attenuates liver inflammation and fibrosis [28] [26]. Furthermore, both canonical and non-canonical inflammasome pathways have been implicated in MASH development [29,30]. Collectively, these findings suggest that the role of NLRP3 in MASH is highly context dependent and may vary according to dietary model, disease stage, and cell specificity.

Hepatic *Timp1* expression was significantly increased in CDAHFD-fed mice, supporting activation of fibrogenic responses in this model. In contrast, *Mmp2* and *Mmp7* expression levels showed only trends toward increased expression. Because matrix metalloproteinases are dynamically regulated according to the stage of liver injury and extracellular matrix remodeling, the absence of significant changes in these genes does not necessarily contradict the presence of hepatic fibrosis [31]. Rather, the concurrent increase in *Timp1* together with Sirius Red staining and *Col1a1* expression supports the establishment of hepatic fibrogenesis in the present model. Importantly, these fibrosis-associated markers did not differ between WT and NLRP3-KO mice, indicating that genetic deletion of NLRP3 was insufficient to modify extracellular matrix remodeling or fibrogenic responses under CDAHFD-fed conditions.

Interestingly, hepatic *Il1b* expression tended to be lower in NLRP3-KO mice, whereas *Il18* expression remained unchanged. These findings suggest that genetic deletion of NLRP3 may partially attenuate inflammatory signaling, but this reduction was insufficient to alter overall hepatic inflammation, fibrosis, or steatosis under the present experimental conditions. The lack of significant differences in downstream pathological outcomes further indicates that inflammatory pathways independent of NLRP3 may substantially contribute to disease progression in the CDAHFD-induced MASH model. The absence of significant changes in *Il18* expression is not unexpected because IL-18 can be regulated through inflammasome-independent mechanisms and its biological activity is primarily determined by post-translational processing rather than transcript abundance [32,33].

Several limitations should be considered. First, the sample size in each group was small, which may have limited statistical power to detect subtle but biologically relevant differences. Although no significant differences were observed in major histological and biochemical outcomes, the possibility of type II error cannot be excluded. The modest reduction in *Adgre1* expression suggests that NLRP3 deletion may exert measurable but relatively small effects, which could require larger cohorts to confirm. Second, only systemic knockout mice were evaluated; cell-specific or temporally controlled inhibition may yield different outcomes. Third, activation of the NLRP3 inflammasome was not directly evaluated by measuring downstream inflammasome signaling such as caspase-1 activation or IL-1β maturation; therefore, the precise contribution of inflammasome activity requires further investigation. Finally, only the CDAHFD model was evaluated. Since the contribution of NLRP3 may differ according to dietary models and disease stages, validation in additional experimental models will be required to determine the generalizability of the present findings.

Our findings are consistent with recent reports demonstrating that systemic NLRP3 deficiency does not substantially ameliorate diet-induced MASH. Rather than contradicting these previous studies, our results independently reproduce these observations in the CDAHFD model, thereby strengthening the evidence that global NLRP3 deficiency alone is insufficient to prevent MASH progression across different dietary models. Further studies are required by focusing on cell-specific targeting strategies and context-dependent inflammasome regulation.

## CRediT authorship contribution statement

**Daisuke Taguchi:** Investigation. **Yohei Shirakami:** Writing – review & editing, Conceptualization. **Toshihide Maeda:** Investigation. **Daisuke Minowa:** Investigation. **Masaya Kubota:** Investigation. **Hiroyasu Sakai:** Investigation. **Hiroyuki Tomita:** Investigation. **Masahito Shimizu:** Supervision, Funding acquisition.

## Ethics declaration

This study was conducted in accordance with the ARRIVE (Animal Research: Reporting of In Vivo Experiments) guidelines. This study was approved by the Institutional Animal Experiments Committee of Gifu University. (Approval No. AG-P-C-20230011)

## Patient consent statement

None.

## Permission to reproduce material from other sources

None.

## Clinical trial registration

None.

## Ethics approval statement

The study protocol was reviewed and approved by the Institutional Animal Experiments Committee of Gifu University (approval number: AG-P-C-20230011).

## Declaration of generative AI and AI-assisted technologies in the manuscript preparation process

During the preparation of this work the authors used the ChatGPT (OpenAI) in order to refine English. After using this tool/service, the authors reviewed and edited the content as needed and takes full responsibility for the content of the published article.

## Funding statement

This work was supported by the 10.13039/501100001691JSPS KAKENHI (grant number JP23K07433).

## Declaration of competing interest

The authors declare that they have no known competing financial interests or personal relationships that could have appeared to influence the work reported in this paper.

## Data Availability

Data will be made available on request.
